# Supplementary material for: Exon skipping induces uniform dystrophin rescue with dose-dependent restoration of serum miRNA biomarkers and muscle biophysical properties
Source: Mol Ther Nucleic Acids. 2022 Aug 25;29:955–68. doi: 10.1016/j.omtn.2022.08.033 (PMC9464767; doi:10.1016/j.omtn.2022.08.033)
Supplement: Document S1. Figures S1–S3 and Tables S1–S5 [file mmc1.pdf]

OMTN, Volume *29*

Supplemental information

Exon skipping induces uniform dystrophin rescue with dose-dependent restoration of serum miRNA biomarkers and muscle biophysical properties

Katarzyna Chwalenia, Jacopo Oieni, Joanna Zemła, Małgorzata Lekka, Nina Ahlskog, Anna M.L. Coenen-Stass, Graham McClorey, Matthew J.A. Wood, Yulia Lomonosova, and Thomas C. Roberts

Table S1

Literature analysis of miRNA RT-qPCR detection technologies.

First Author (Year)	Title	TaqMan	miRCURY	miScript	miRcute	NCode VILO	ReverTra Ace	TaqMan Advanced
**Yuasa** (2008)	MicroRNA-206 Is Highly Expressed in Newly Formed Muscle Fibers: Implications Regarding Potential for Muscle Regeneration and Maturation in Muscular Dystrophy	■						
**Greco** (2009)	Common micro-RNA signature in skeletal muscle damage and regeneration induced by Duchenne muscular dystrophy and acute ischemia	■						
**Cacchiarelli** (2010)	MicroRNAs involved in molecular circuitries relevant for the Duchenne muscular dystrophy pathogenesis are controlled by the dystrophin/nNOS pathway	■						
**Nakasa** 2010	Acceleration of muscle regeneration by local injection of muscle-specific microRNAs in rat skeletal muscle injury model	■						
**Alexander** (2011)	Regulation of DMD pathology by an ankyrin-encoded miRNA	■						
**Cacchiarelli** (2011)	miRNAs as serum biomarkers for Duchenne muscular dystrophy			■				
**Cacchiarelli** (2011)	miR-31 modulates dystrophin expression: new implications for Duchenne muscular dystrophy therapy			■				
**Koutalianos** (2011)	Expression of miR-1, miR-133a, miR-133b and miR-206 increases during development of human skeletal muscle	■						
**Mizuno** (2011)	Identification of muscle-specific microRNAs in serum of muscular dystrophy animal models: promising novel blood-based markers for muscular dystrophy	■						
**Cheung** (2012)	Maintenance of muscle stem-cell quiescence by microRNA-489	■						
**Goyenvalle** (2012)	Rescue of severely affected dystrophin/utrophin-deficient mice through scAAV-U7snRNA-mediated exon skipping	■						
**Roberts** (2012)	Expression analysis in multiple muscle groups and serum reveals complexity in the microRNA transcriptome of the mdx mouse with implications for therapy	■						
**Wang** (2012)	Loss of miR-29 in Myoblasts Contributes to Dystrophic Muscle Pathogenesis	■						
**Roberts** (2013)	Extracellular microRNAs are dynamic non-vesicular biomarkers of muscle turnover	■	■					
**Vignier** (2013)	Distinctive serum miRNA profile in mouse models of striated muscular pathologies	■	■					
**Zaharieva** (2013)	Dystromirs as serum biomarkers for monitoring the disease severity in Duchenne muscular Dystrophy	■						
**Alexander** (2014)	MicroRNA-486–dependent modulation of DOCK3/PTEN/AKT signaling pathways improves muscular dystrophy–associated symptoms	■						
**Fortere** (2014)	Myotube-derived exosomal miRNAs downregulate Sirtuin1 in myoblasts during muscle cell differentiation	■						
**Gomes** (2014)	Circulating miR-1, miR-133a, and miR-206 levels are increased after a half-marathon run	■						
**Holmberg** (2014)	Laminin α2 chain-deficiency is associated with microRNA deregulation in skeletal muscle and plasma		■					
**Hu** (2014)	Serum miR-206 and other muscle-specific microRNAs as non-invasive biomarkers for Duchenne muscular dystrophy						■	
**Jeanson-leh** (2014)	Serum profiling identifies novel muscle miRNA and cardiomyopathy-related miRNA biomarkers in Golden Retriever muscular dystrophy dogs and Duchenne muscular dystrophy patients	■	■					
**Li** (2014)	Circulating Muscle-specific miRNAs in Duchenne Muscular Dystrophy Patients				■			
**Matsuzaka** (2014)	Three novel serum biomarkers, miR-1, miR-133a, and miR-206 for Limb-girdle muscular dystrophy, Facioscapulohumeral muscular dystrophy, and Becker muscular dystrophy	■						
**Roberts** (2014)	Assessment of RT-qPCR normalization strategies for accurate quantification of extracellular microRNAs in murine serum	■						

First Author (Year)	Title	TaqMan	miRCURY	miScript	miRcute	NCode VILO	ReverTra Ace	TaqMan Advanced
**Toivonen** (2014)	MicroRNA-206: A Potential Circulating Biomarker Candidate for Amyotrophic Lateral Sclerosis	■						
**Betts** (2015)	Prevention of exercised induced cardiomyopathy following Pip-PMO treatment in dystrophic mdx mice	■						
**Fiorillo** (2015)	TNF-α-induced microRNAs control dystrophin expression in Becker muscular dystrophy	■						
**Koutalianos** (2015)	MyoD transcription factor induces myogenesis by inhibiting Twist-1 through miR-206	■						
**Koutalianos** (2015)	Elevated Muscle-Specific miRNAs in Serum of Myotonic Dystrophy Patients Relate to Muscle Disease Progress	■						
**Roberts** (2015)	Multi-level omics analysis in a murine model of dystrophin loss and therapeutic restoration	■						
**Zanotti** (2015)	Opposing roles of miR-21 and miR-29 in the progression of fibrosis in Duchenne muscular dystrophy			■				
**Anya-Segura** (2016)	Serum Levels of MicroRNA-206 and Novel Mini-STR Assays for Carrier Detection in Duchenne Muscular Dystrophy	■						
**Becker** (2016)	Identification of cardiomyopathy associated circulating miRNA biomarkers in patients with muscular dystrophy using a complementary cardiovascular magnetic resonance and plasma profiling approach	■						
**Coenen-Stass** (2016)	Selective release of muscle-specific, extracellular microRNAs during myogenic differentiation	■						
**Isareli** (2016)	Circulating miRNAs are generic and versatile therapeutic monitoring biomarkers in muscular dystrophies		■					
**Matsuzaka** (2016)	Characterization and Functional Analysis of Extracellular Vesicles and Muscle-Abundant miRNAs (miR-1, miR-133a, and miR-206) in C2C12 Myocytes and mdx Mice	■						
**Perfetti** (2016)	Plasma microRNAs as biomarkers for myotonic dystrophy type 1	■						
**Perfetti** (2016)	Validation of plasma microRNAs as biomarkers for myotonic dystrophy type 1	■						
**Robriquet** (2016)	Identification in GRMD dog muscle of critical miRNAs involved in pathophysiology and effects associated with MuStem cell transplantation	■						
**Coenen-Stass** (2017)	Comprehensive RNA-sequencing analysis in serum and muscle reveals novel small RNA signatures with biomarker potential for DMD	■						
**Fry** (2017)	Myogenic Progenitor Cells Control Extracellular Matrix Production by Fibroblasts during Skeletal Muscle Hypertrophy			■				
**Koutsoulidou** (2017)	Identification of exosomal muscle-specific miRNAs in serum of myotonic dystrophy patients relating to muscle disease progress	■						
**Llano-Diez** (2017)	Digital PCR quantification of miR-30c and miR-181a as serum biomarkers for Duchenne muscular dystrophy	■						
**Catapano** (2018)	Altered Levels of MicroRNA-9, -206, and -132 in Spinal Muscular Atrophy and Their Response to Antisense Oligonucleotide Therapy	■						
**Catapano** 2018	Downregulation of miRNA-29, -23 and -21 in urine of Duchenne muscular dystrophy patients		■					■
**D'Agostino** (2018)	Role of miR-200c in Myogenic Differentiation Impairment via p66Shc: Implication in Skeletal Muscle Regeneration of Dystrophic mdx Mice	■						
**Fiorillo** (2018)	Muscle miRNAome shows suppression of chronic inflammatory miRNAs with both prednisone and vamorolone	■						
**Florian** (2018)	Identification of Cardiomyopathy-Associated Circulating miRNA Biomarkers in Muscular Dystrophy Female Carriers Using a Complementary Cardiac Imaging and Plasma Profiling Approach	■						
**Guilbaud** (2018)	miR-708-5p and miR-34c-5p are involved in nNOS regulation in dystrophic context	■	■					
**Kozakowska** (2018)	Lack of Heme Oxygenase-1 Induces Inflammatory Reaction and Proliferation of Muscle Satellite Cells after Cardiotoxin-Induced Skeletal Muscle Injury					■		
**Pietraszek-Gremplewicz** (2018)	Heme Oxygenase-1 Influences Satellite Cells and Progression of Duchenne Muscular Dystrophy in Mice		■					
**Zanotti** (2018)	Exosomes and exosomal miRNAs from muscle-derived fibroblasts promote skeletal muscle fibrosis			■				

First Author (Year)	Title	TaqMan	miRCURY	miScript	miRcute	NCode VILO	ReverTra Ace	TaqMan Advanced
**Coenen-Stass** (2019)	Extracellular microRNAs exhibit sequence-dependent stability and cellular release kinetics	■						
**Morgoulis** (2019)	sPIF promotes myoblast differentiation and utrophin expression while inhibiting fibrosis in Duchenne muscular dystrophy via the H19/miR-675/let-7 and miR-21 pathways	■						
**Verma** (2019)	Inhibition of microRNA-92a increases blood vessels and satellite cells in skeletal muscle but does not improve duchenne muscular dystrophy-related phenotype in mdx mice			■				
**Catapano** (2020)	Novel free-circulating and extracellular vesicle-derived miRNAs dysregulated in Duchenne muscular dystrophy	■	■					
**Kinder** (2020)	Muscle Weakness in Myositis: MicroRNA-Mediated Dystrophin Reduction in a Myositis Mouse Model and Human Muscle Biopsies	■						
**Mousa** (2020)	Circulating MicroRNAs in Duchenne Muscular Dystrophy			■				
**Sandona** (2020)	HDAC inhibitors tune miRNAs in extracellular vesicles of dystrophic muscle-resident mesenchymal cells	■						
**Trifunov** (2020)	Longitudinal Study of Three microRNAs in Duchenne Muscular Dystrophy and Becker Muscular Dystrophy	■						
**van Westering** (2020)	Uniform sarcolemmal dystrophin expression is required to prevent extracellular microRNA release and improve dystrophic pathology	■						
**van Westering** (2020)	Mutation-independent proteomic signatures of pathological progression in murine models of Duchenne muscular dystrophy	■						
**Zhang** (2020)	Serum MyomiRs as Biomarkers for Female Carriers of Duchenne/Becker Muscular Dystrophy				■			
**Dobrowolny** (2021)	A longitudinal study defined circulating microRNAs as reliable biomarkers for disease prognosis and progression in ALS human patients		■					
**Hightower** (2021)	miR-486 is an epigenetic modulator of Duchenne muscular dystrophy pathologies	■						
**Mytidou** (2021)	Muscle-derived exosomes encapsulate myomiRs and are involved in local skeletal muscle tissue communication	■						

Table S2

Oligonucleotides used in this study.

ID	Sequence (5' to 3')
**Exon skipping oligonucleotides (PMO)**	
Dmd **Ex23 skipping**	GGCCAAACCTCGGCTTACCTGAAAT
**miRNA mimics/spikes (RNA)**	
**mmu-miR-1a-3p**	UGGAAUGUAAAGAAGUAUGUAU
**mmu-miR-133a-3p**	UUUGGUCCCCUUCAACCAGCUG
**mmu-miR-133b-3p**	UUUGGUCCCCUUCAACCAGCUA
**mmu-miR-206-3p**	UGGAAUGUAAGGAAGUGUGUGG
**cel-miR-39**	UCACCGGGUGUAAAUCAGCUUG

Table S3

Antibodies used in this study.

Target protein	Host	Product ID	Manufacturer	Dilution
**Dystrophin (DMD)** for WB	Mouse mAb	NCL-DYS1	Leica Biosystems	1:100
**Vinculin (VCL)**	Mouse mAb	V9131	Sigma-Aldrich/Merck	1:200
**Anti-mouse IgG, HRP-linked**	Horse	7076	Cell Signaling Technology	1:10,000
**Dystrophin (DMD)** for IF	Rabbit pAb	ab15277	Abcam	1:1,000
**Laminin subunit alpha 2 (LAMA2)**	Rat mAb	L0663 (clone: 4H8-2)	Sigma-Aldrich/Merck	1:1,000
**Anti-rabbit IgG Alexa Fluor 594**	Goat	ab150080	Abcam	1:500
**Anti-rat IgG Alexa Fluor 488**	Goat	ab150157	Abcam	1:500

Table S4

List of primer sequences used in this study.

Exon skipping RT-qPCR assay probes include 5′ terminal fluorophores (either FAM or HEX), 3′-terminal Iowa Black fluorescence quencher, and contain internal ZEN modifications. Primer probe assays were obtained from IDT.

ID	Sequence (5' to 3')
**Exon Skipping RT-qPCR**	
**qExon22-24-Fwd**	CTGAATATGAAATAATGGAGGAGAGACTCG
**qExon22-24-Rev**	CTTCAGCCATCCATTTCTGTAAGGT
**qExon22-24-Probe**	/5FAM/ATGTGATTC/ZEN/TGTAATTTCC/3IABkFQ/
**qExon23-24-Fwd**	CAGGCCATTCCTCTTTCAGG
**qExon23-24-Rev**	GAAACTTTCCTCCCAGTTGGT
**qExon23-24-Probe**	/5HEX/TCAACTTCA/ZEN/GCCATCCATTTCTGTAAGGT/3IABkFQ/

Table S5

List of TaqMan assays used in this study.

All products were obtained from Thermo Fisher Scientific.

Target	Product ID	Detection Channel
**Small RNA TaqMan RT-qPCR**		
mmu-miR-1a-3p	002222	FAM
mmu-miR-133a-3p	002246	FAM
mmu-miR-206-3p	000510	FAM
mmu-miR-483-3p	002560	FAM
cel-miR-39	000200	FAM

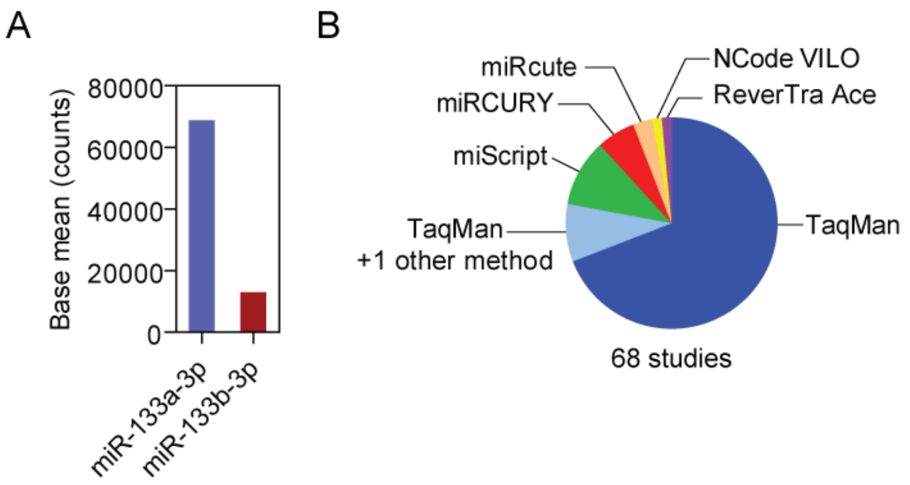

Figure S1

Analysis of miR-133a/b expression and miRNA detection method usage.

(**A**) Small RNA-seq data for mouse muscle (WT, *mdx*, and PPMO-treated *mdx*). Base mean values (i.e. the average across all samples) are shown. These data show that miR-133b-3p is expressed at much lower levels than miR-133a-3p. Full dataset was published as Coenen-Stass *et al.* [17] and are publicly available (SRA project identifier: SRP102619). (**B**) Analysis of miRNA RT-qPCR methodologies utilized in 68 studies related to miRNA biomarkers in DMD, other myopathies, and muscle biology more generally. The Small RNA TaqMan method is by far the most commonly used technology. Full details of this analysis are in **Table S5**.

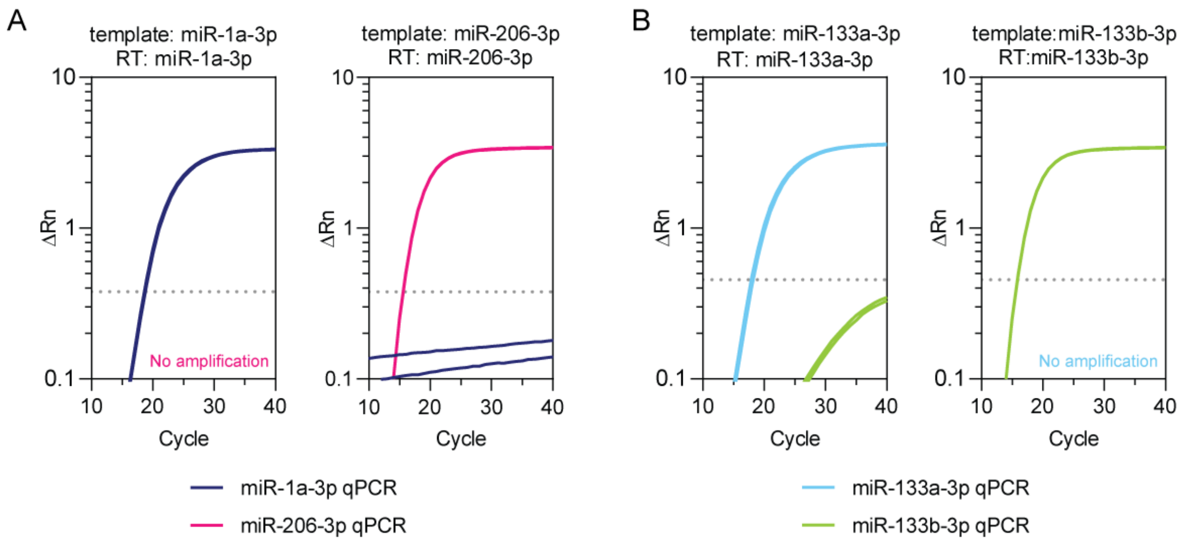

Figure S2

Small RNA TaqMan assays can discriminate between closely-related cDNA templates.

Artificial samples containing 50 fmol of synthetic miRNA mimic oligonucleotide templates were reverse transcribed using the on-target RT primer, and then amplified using either the on-target TaqMan assay or the off-target TaqMan assay for the closely-related miRNA. Amplification plots are shown for (**A**) miR-1a-3p and miR-206-3p, and (**B**) miR-133a-3p and miR-133b-3p. The threshold is indicated by a dotted line. When reactions were performed in this manner, there was either no amplification for the off-target assays or the amplification curves failed to cross the threshold within 40 cycles.

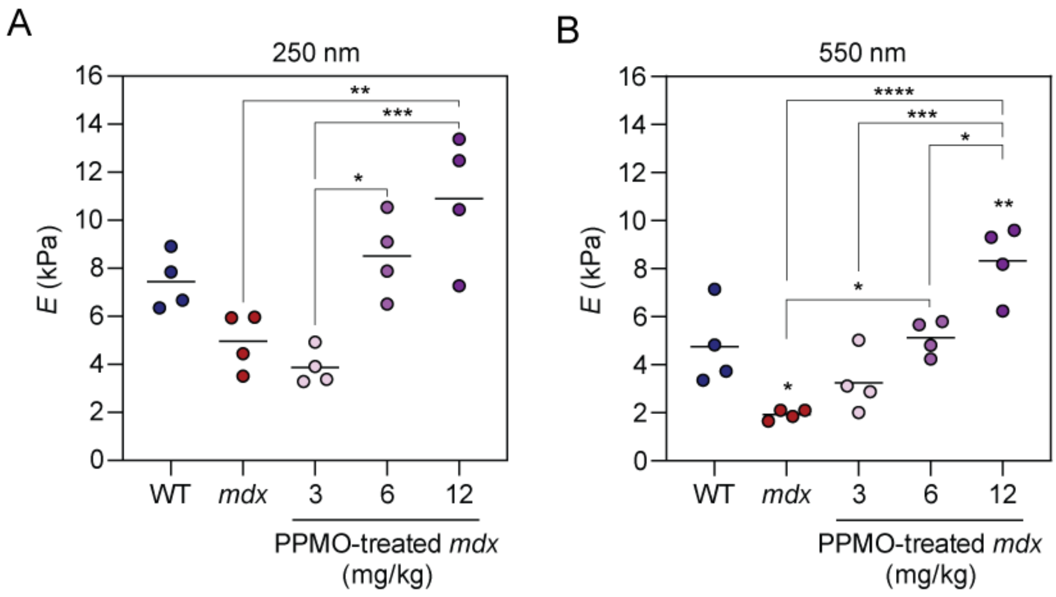

Figure S3

Dose-dependent changes in muscle stiffness following exon skipping treatment.

Atomic force microscopy was performed in order to determine Young's modulus (*E*) calculated for indentation depth depths of (**A**) 250 nm and (**B**) 550 nm, in TA muscle explants from WT, *mdx*, and PPMO-treated *mdx* mice. Mean values and individual sample data points are shown. Statistical significance was tested by one-way ANOVA and Bonferroni *post hoc* test. Statistical comparisons are made to the WT control group unless otherwise indicated, $*p < 0.05$, $**p < 0.01$, $***p < 0.001$, $****p < 0.0001$.
